# Effect of Near Work on Intraocular Pressure in Emmetropes

**DOI:** 10.1155/2020/1352434

**Published:** 2020-01-28

**Authors:** Aaron Z. Priluck, Aidan B. Hoie, Robin R. High, Vikas Gulati, Deepta A. Ghate

**Affiliations:** ^1^University of Nebraska Medical Center, College of Medicine, Omaha, NE, USA; ^2^University of Nebraska Medical Center, College of Public Health, Omaha, NE, USA; ^3^University of Nebraska Medical Center, Stanley M. Truhlsen Eye Institute, Omaha, NE, USA

## Abstract

**Objective:**

To determine whether accommodation induced by reading alters intraocular pressure (IOP) in healthy, young, emmetropic adults and to document the duration and magnitude of this effect.

**Design:**

Cross-sectional study. *Participants*. Fifteen healthy, emmetropic young adults.

**Methods:**

Subjects performed 20 minutes of near work (reading at 33 cm) followed by 20 minutes of far work (reading at 520 cm) while IOP was measured using an iCare tonometer at baseline and every 5 minutes thereafter. Statistical analysis was performed using repeated measures ANOVA. *Main Outcome Measures*. Intraocular pressure.

**Results:**

IOP decreased significantly compared to baseline IOP after 10 minutes of near work (average change of −1.60 ± 2.2 (SD) mm Hg, *p* < 0.05). IOP remained lower than baseline IOP throughout all subsequent near and far work. The difference in IOP at the end of experimentation compared to baseline IOP was −1.87 ± 1.81 mm Hg (*p* < 0.05). IOP remained lower than baseline IOP throughout all subsequent near and far work. The difference in IOP at the end of experimentation compared to baseline IOP was −1.87 ± 1.81 mm Hg (

**Conclusions:**

Near work decreases IOP in healthy emmetropes, and this effect is sustained for at least 20 minutes after discontinuing prolonged near work. Providers may need to consider this effect when measuring IOP in clinical practice.

## 1. Introduction

Glaucoma is the most common cause of irreversible blindness in the world [1, 2]. The only known modifiable risk factor for glaucoma is intraocular pressure (IOP), making accurate monitoring of IOP critical to successful disease management [3, 4]. Accommodative effort has been shown to decrease IOP in a diverse range of participants including healthy subjects [5–8], glaucoma patients [9], and both near and farsighted participants [10, 11]. Accommodation may decrease IOP via the contraction of ciliary muscle and the opening of Schlemm's canal, which leads to increased aqueous outflow through the conventional pathway [12, 13].

Although the effect of accommodation on IOP has been well studied, the duration of these changes has not. The purpose of our study was to measure both the magnitude and duration of accommodation-induced IOP changes during near and far work. We hypothesized that accommodation would reduce IOP, and that this reduction would be sustained after cessation of near work.

## 2. Materials and Methods

This was a cross-sectional study conducted at the University of Nebraska Medical Center (UNMC) and was approved by the institutional review board. Informed consent was obtained from all subjects, and the tenets of the Declaration of Helsinki were adhered to. Subjects were included if they were between 19 and 40 years old and had uncorrected visual acuity of at least 20/20 in both eyes. Subjects were excluded if they had any history of ocular disease or surgery or took medications known to alter IOP. Visual acuity was measured using the Snellen chart monocularly and binocularly, and autorefraction was performed on all subjects. Basic demographic information on all participants was collected including age, race, and gender.

Fifteen subjects performed 20 minutes of near work followed by 20 minutes of far work using both eyes together. IOP was measured in the right eye at baseline and every five minutes thereafter, leading to a total of nine IOP measurements. During each IOP measurement, all participants continued performing near or far work by using their left eye while their right eye's line of sight was interrupted by the tonometer. All IOP measurements were performed using an iCare TA01i rebound tonometer (Icare USA, Raleigh, NC, USA) by trained personnel (AP, AH). Experimentation was performed during daylight hours for all subjects, and the protocol was completed for each subject without any pauses during experimentation.

**Figure 1:**
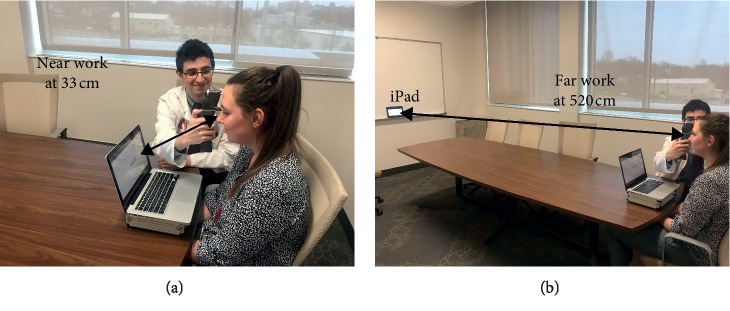
IOP measurements during near (a) and far (b) work.

Near work consisted of reading the story “The Most Dangerous Game” by Richard Connell word by word from a 2011 MacBook Pro laptop (Apple Inc., USA) placed at a distance of 33 cm (visual angle of 1.9°) at 150 words per minute as shown in Figure 1. The free online software Squirt was used to play the story word by word, enabling participants to maintain fixation on the same spot while reading (Cameron Boehmer, USA) [14].

Far work consisted of reading the same story but on a 3^rd^ generation 9.7 inch iPad (Apple Inc., USA) from a distance of 520 cm (visual angle 0.77°) as shown in Figure 1. The near and far stories were synchronized in time so that when participants switched between screens (i.e., switched from near to far work at 20 minutes), they were at the same point in the story. The brightness settings of both the laptop and tablet were maximized, and the story duration was 40 minutes.

For statistical analysis, the outcome variable was defined as the difference of the IOP measurement at each time point from the baseline (time = 0 minutes) IOP for each subject. A repeated measures ANOVA was used to account for the correlation structure of the data collected from each subject over time. The differences were evaluated with time as a fixed effect. The baseline measurement served as a covariate in the model. An autoregressive correlation structure was applied for analysis. Least square means of the differences and their confidence intervals at each time point indicate where the average changes from baseline at each time point do not equal 0. To account for multiplicity of comparisons, *p* values and confidence intervals for the differences were adjusted using simulation, the method recommended for repeated measures ANOVAs [15]. The MIXED procedure from SAS/STAT software, Version 9.4 (© 2002–2012), of the SAS System for Windows (Cary, NC) was used for all calculations.

## 3. Results

**Figure 2:**
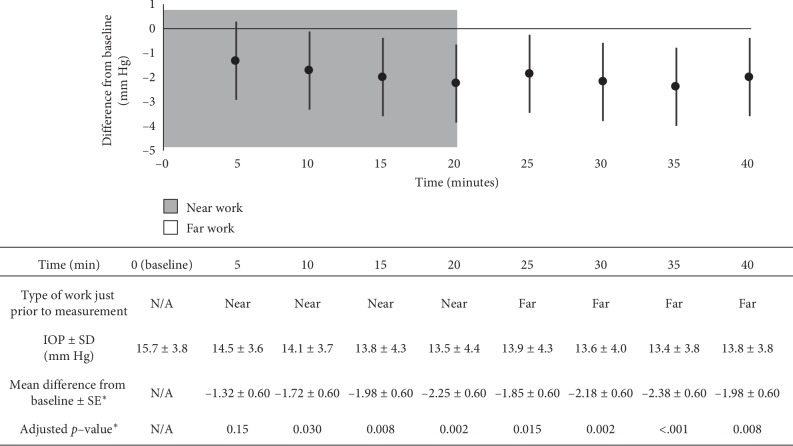
Graphed statistical results with raw data in table (statistically significant differences exist when the graphed 95% confidence interval does not contain zero, which corresponds to *p* values less than 0.05 (bold); asterisk indicates results derived from the repeated measures ANOVA).

All 15 participants successfully completed the study. The cohort consisted of 12 males and 3 females who were on average 24.3 ± 1.3 (SD) years old. All participants were Caucasian and had uncorrected visual acuity of at least 20/20. Spherical equivalent by autorefraction across all right eyes ranged from −0.875 to 0.125 Diopters (D), averaging −0.450 D.  Figure 2 shows the IOP measurements and statistical results; IOP was statistically significantly less than baseline IOP beginning at 10 minutes through the remainder of the experiment.

IOP decreased by 2.1 ± 2.6 (SD) mm Hg after 20 minutes of accommodation (*p*=0.002 compared to baseline IOP) followed by a subsequent rise of only 0.30 ± 1.5 mm Hg after the subsequent 20 minutes of far work (*p*=0.008 compared to baseline). 13 of 15 subjects (87%) had an IOP of at least 2 mm Hg less than their baseline IOP at some point during experimentation. Similarly, 9/15 (60%) participants had IOPs of at least 4 mm Hg lower than baseline at some point during experimentation. Only 3 of 15 subjects (20%) had an IOP of at least 2 mm Hg greater than their baseline IOP at some point during experimentation. The average difference between baseline IOP and minimum IOP during experimentation was −3.8 ± 2.2 mm Hg (range: 0 to −8.0 mm Hg).

## 4. Discussion

Accommodation reduced IOP, and this reduction was sustained for a period of at least 20 minutes following 20 minutes of accommodation. Note that Figure 2 demonstrates the relationship of near work causing IOP to decrease and that IOP continues to remain significantly lower than baseline IOP throughout the 20 minutes of far work. Prior studies examining the effect of accommodation on IOP are summarized in Table 1. Note that unlike our study, none of these prior studies measured IOP after relaxation of accommodation except for one, which did not make a conclusion regarding the duration of IOP changes during accommodation [5].

The studies summarized in Table 1 mostly consisted of healthy young adults with the exceptions of Armaly et al. who studied a population of 45–65 years and Cassidy et al. who studied IOP changes in glaucoma subjects [5, 9]. Two studies found no statistically significant IOP lowering with accommodation in emmetropes [11, 17]. Notably however, these studies used lenses rather than near work to induce accommodation. It is well established that near work induces the triad of accommodation, convergence, and miosis [18]. However, miosis does not co-occur with accommodation when mild accommodation is induced in people with considerable accommodative reserve [19]. Thus, the probable lack of miosis in their participants, given that a lens was used for accommodation induction, may explain the minimal and statistically insignificant IOP changes and suggests that miosis is critical to the underlying mechanism of IOP decreasing during near work. Moreover, both of those studies were performed in China, whereas our participants were all Caucasian.

We demonstrated that IOP does not quickly rebound to baseline after near work. This is possibly due to the increased efflux of aqueous humor out of the anterior chamber followed by a finite amount of time necessary for its regeneration. The approximately 2 mm Hg decrease in IOP from baseline after 20 minutes of near work that was found corresponds to a 2 to 3 *μ*L loss of aqueous humor based on the Goldmann equation. The amount of time necessary to retain an additional 2 to 3 *μ*L of aqueous humor during far work would depend on how rapidly the outflow capacity decreased back to its baseline. In addition, the rate of aqueous formation could possibly be changing during experimentation, which further confounds the expected rate of return to baseline IOP. However, both pilocarpine and accommodation lead to muscarinic stimulation, and pilocarpine is known to alter outflow but not aqueous production [12, 13, 20]. Fluorophotometry and tonography are necessary to better understand the mechanism underlying our results. A better understanding of this mechanism may elucidate potential drug targets and reveal why accommodation seems to alter some participants' IOPs more than others.

One limitation of our study is that only one iCare reading was taken per time point. This was done to avoid corneal injury and limit accommodative disruption given that IOP was measured at many time points. However, the iCare has been shown to be a reliable and robust way to measure IOP [21–24]. In addition, despite finding statistically significant results, our sample size was small. Moreover, all of our study participants were healthy, emmetropic young adults, and our results cannot be extended to all patients. Future studies with participants who have pathologic ocular conditions such as pseudophakia and presbyopia are desirable.

In conclusion, our study corroborates that near work decreases IOP and supports the novel finding that this decrease in IOP is sustained even when near work has ended. Patients performing near work (e.g., reading or using a cellphone) in waiting rooms will artefactually lower their IOP measurements. This may lead to undertreatment of IOP. Moreover, this effect may prove to be therapeutically useful in some patients.

## Figures and Tables

**Table 1:** Summary of prior studies examining the effect of accommodation on intraocular pressure; BCVA: best corrected visual acuity.

Study	Participants	Accommodative task	Outcome measures	Tonometry method	Main result
Armaly and Rubin [5]	10 healthy subjects with best corrected visual acuity (BCVA) of at least 20/20 divided into two age groups (20–25 and 45–55 years)	View a target at 25 cm with variable lenses to induce variable magnitudes of accommodation	Minute-by-minute IOP measurements with variable durations of accommodation and relaxation	Goldmann applanation	Younger and older age groups had mean maximum reductions in IOP of 4.5 mm Hg and 2.3 mm Hg, respectively

Mauger et al. [8]	30 young (aged 22 to 35 years), healthy subjects with BCVA of at least 20/20 divided into three groups (mean age not reported)	View a single row of 6/6 letters with either no lens (group 1), a −4.0 D lens (group 2), or a −1.5 D lens (group 3) added at 2.5 minutes to induce varying amounts of accommodation	IOP at baseline and at 3 and 6 minutes	Goldmann applanation	At 3 and 6 minutes, groups 1–3 had respective IOP changes of 0 and 0.35 mm Hg, −1.32 and −2.38 mm Hg, and −1.15 and −2.15 mm Hg

Cassidy et al. [9]	20 volunteers with newly diagnosed primary open angle glaucoma divided into two groups (mean age of 66 years)	Read for ten minutes	Group A: IOP at baseline and after 10 minutes of reading Group B: IOP at baseline and after 10 minutes of watching television at 6 meters	Goldmann applanation	IOP of groups A and B decreased by 2.5 and 0.35 mm Hg, respectively, and IOP of all group A subjects decreased with 9 of 10 decreasing by at least 2 mm Hg

Read et al. [16]	15 myopic and 17 emmetropic healthy adults (mean age of 23 years)	View an n10 size letter to induce 3 D of accommodation	IOP at baseline and after 2 minutes of near work	Pascal dynamic contour tonometer	IOP decreased by 1.8 mm Hg in both groups

Jenssen and Krohn [7]	33 healthy volunteers with BCVA averaging 1.2 (mean age of 24 years)	View Lang fixation cube to induce 3 D of accommodation	IOP at baseline, after 10 minutes of far work, and after 3 minutes of near work (static); on a subsequent day, IOP at baseline and after 3 minutes of rapidly alternating between near and far work (repeated)	Goldmann applanation	IOP decreased from baseline after both static and repeated accommodation with mean reductions in IOP of 1.76 and 2.06 mm Hg, respectively

Yan et al. [17]	46 progressing myopes and 40 emmetropes with BCVA of at least 20/20 (mean age of 24.6 years)	View first-line test object on visual chart at 5 m through 3 D lens until clear; repeat with 6 D lens	IOP at baseline (relaxation of accommodation induced by −3 D lens) and IOP after 3 D and 6 D accommodative tasks; anterior chamber anatomical measurements performed	iCare rebound tonometer	IOP increased in progressing myopes (0.8 and 1.02 mm Hg after 3 and 6 D lenses, respectively) but no significant change in emmetropes; in both, anterior chamber depth and angle decreased and lens thickened

Liu et al. [11]	270 myopes and 48 emmetropes with BCVA of at least 20/20 (mean age of 19.4 years)	View a visual chart at 5 meters with a −3 D lens on subjects' fully corrected lenses	IOP at baseline and after 3 minutes of accommodation, which began after the subject reported subjective resolution of the target after the addition of the −3 D lens	iCare rebound tonometer	No significant IOP change after accommodation in progressing myopes, stable myopes, and emmetropes

## Data Availability

Raw data can be obtained by emailing the corresponding author.
